# Supplementary figures and images for: Single-domain antibody screening by isPLA-seq
Source: Life Sci Alliance. 2021 Oct 21;5(1):e202101115. doi: 10.26508/lsa.202101115 (PMC8548206; doi:10.26508/lsa.202101115)

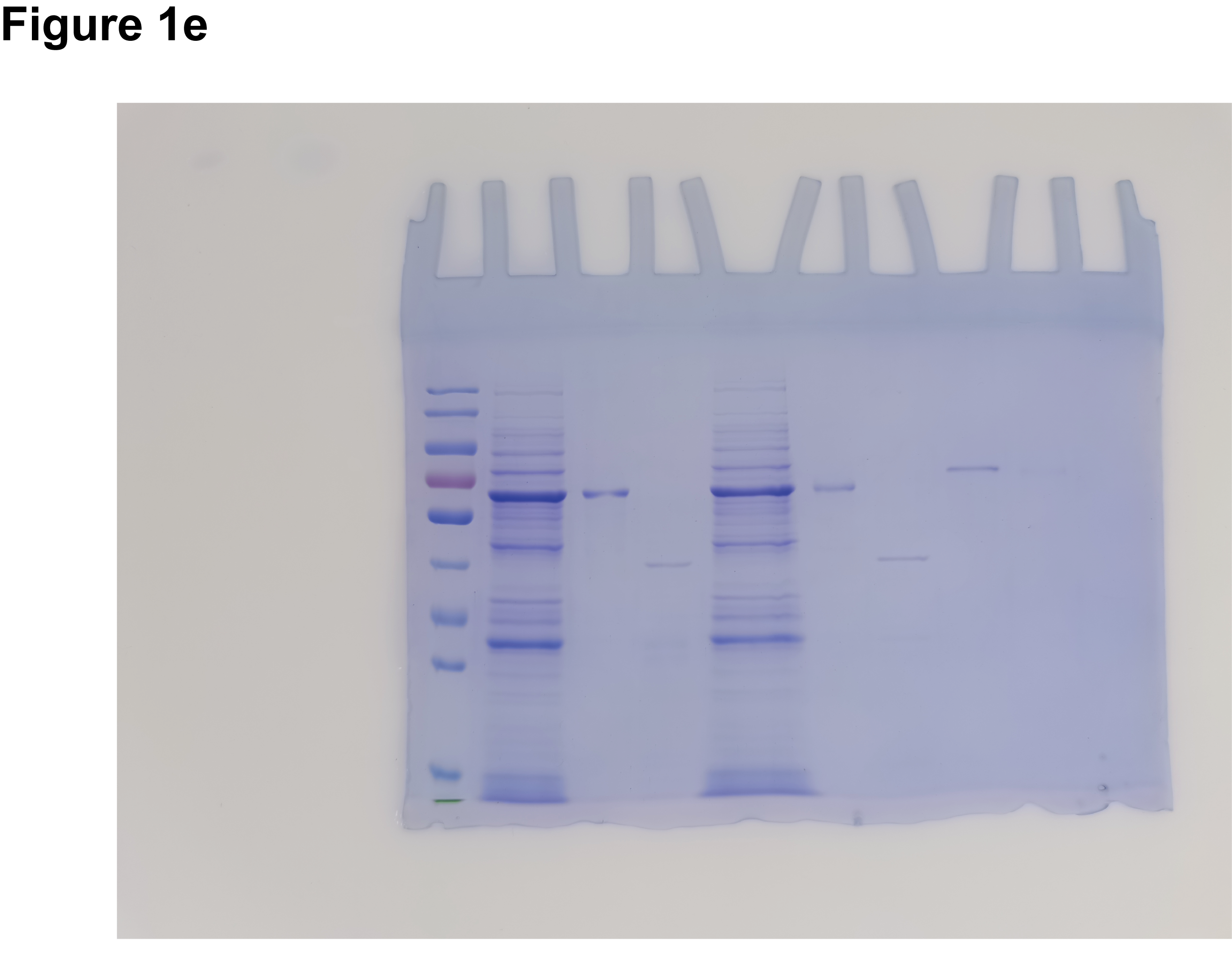

Supplement: Supplementary file 1 [file LSA-2021-01115_SdataF1.tif]

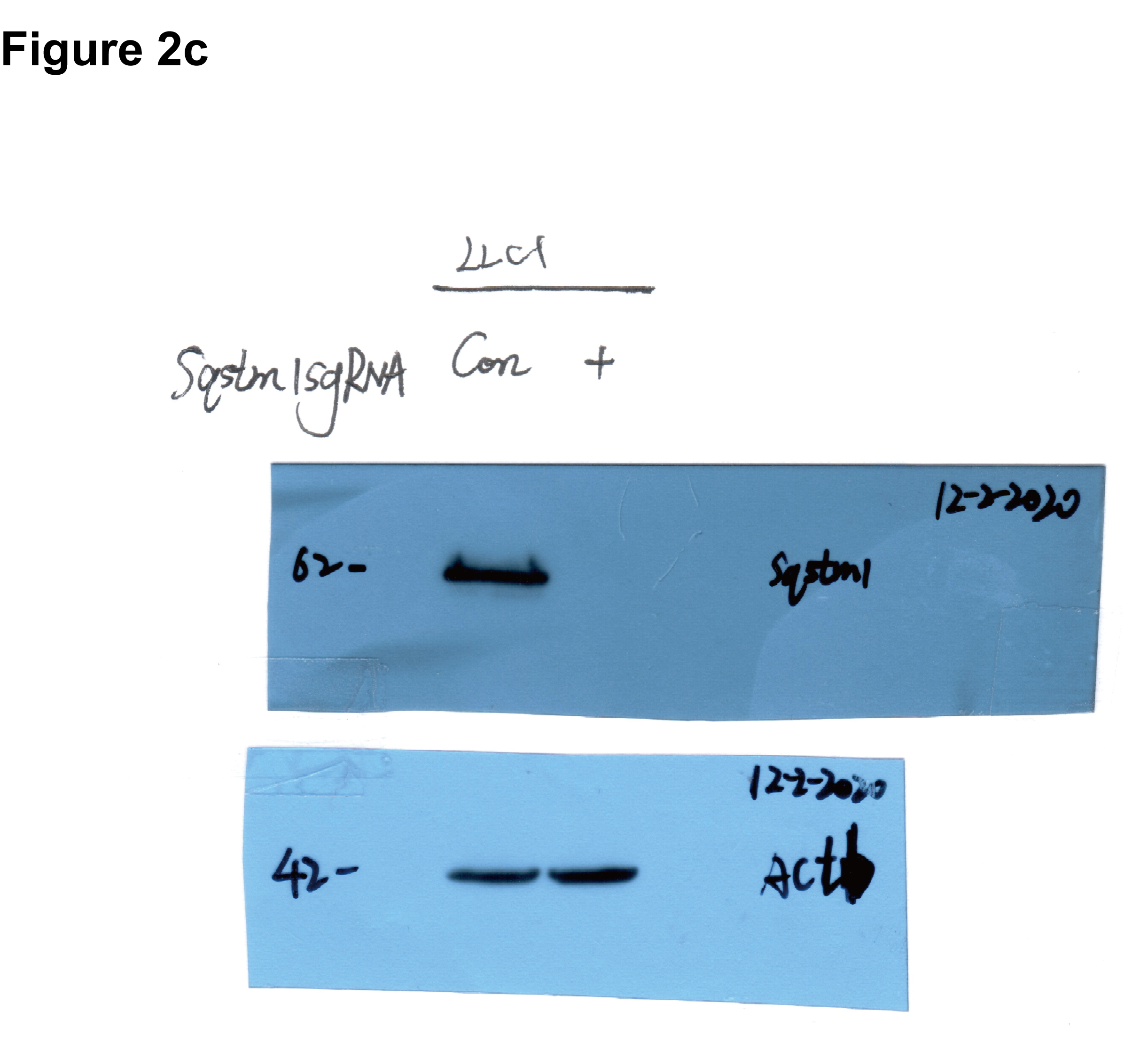

Supplement: Supplementary file 2 [file LSA-2021-01115_SdataF2.tif]

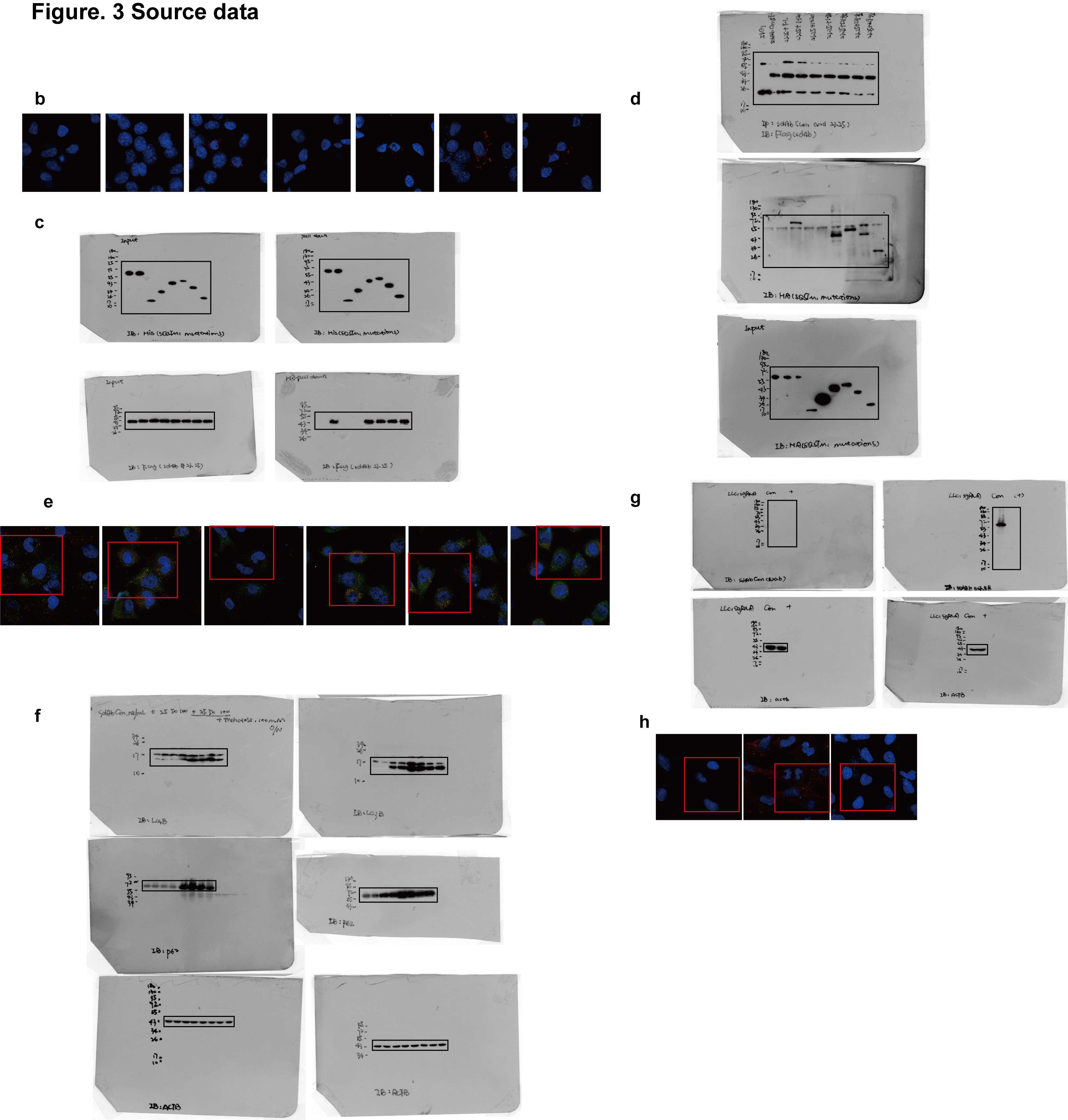

Supplement: Supplementary file 3 [file LSA-2021-01115_SdataF3.tif]
